# Progress and Future Prospectives in Skin-on-Chip Development with Emphasis on the use of Different Cell Types and Technical Challenges

**DOI:** 10.1007/s12015-017-9737-1

**Published:** 2017-05-24

**Authors:** Lenie J. van den Broek, Lambert I. J. C. Bergers, Christianne M. A. Reijnders, Susan Gibbs

**Affiliations:** 1 Department of Dermatology, VU University Medical Center, Amsterdam, The Netherlands; 2 Department of Oral Cell Biology, Academic Center for Dentistry Amsterdam, University of Amsterdam and VU University, Amsterdam, The Netherlands

**Keywords:** Skin equivalent, Skin model, Skin, Cell line, Induced pluripotent stem cell, Organotypic model, Organ-on-chip, Microfluidics, In vitro

## Abstract

Understanding the healthy and diseased state of skin is important in many areas of basic and applied research. Although the field of skin tissue engineering has advanced greatly over the last years, current in vitro skin models still do not mimic the complexity of the human skin. Skin-on-chip and induced pluripotent stem cells (iPSC) might be key technologies to improve in vitro skin models. This review summarizes the state of the art of in vitro skin models with regard to cell sources (primary, cell line, iPSC) and microfluidic devices. It can be concluded that iPSC have the potential to be differentiated into many kinds of immunologically matched cells and skin-on-chip technology might lead to more physiologically relevant skin models due to the controlled environment, possible exchange of immune cells, and an increased barrier function. Therefore the combination of iPSC and skin-on-chip is expected to lead to superior healthy and diseased in vitro skin models.

## Introduction

Skin is an essential and complex barrier in the human body. It has passive functions such as preventing dehydration and it maintains a gradient of gas concentration (O_2_, CO_2_, N_2_). The skin also functions actively by regulating body temperature using sweat glands and hair, it senses heat, pressure and strain and it regulates the composition of the microbiome in conjunction with skin resident dendritic cells. Importantly it also forms a barrier which protects the body against pathogens, UV radiation and penetration of potentially harmful substances. Understanding the healthy and diseased state of skin is important in many areas of basic and applied research ranging from risk assessment of chemicals (including cosmetics) in healthy skin models to eg. fibrosis and tumor disease models. Currently, animal models are extensively used in the preclinical phase of drug development for risk assessment and identifying the mode of action of drugs. However animal models often poorly predict the human response due to differences in skin physiology and immunity [1, 2]. Also current roadmaps for the pharmaceutical and cosmetics industry ask for reduction, refinement and replacement of animals in experiments (7th amendment to EU Cosmetics Directive 76/768/EEC) [3]. Human ex vivo skin explants can be used for risk assessment and drug testing. However, there is little room for manipulation of experimental variables when using such skin biopsies and there are logistical issues with obtaining sufficient samples for experimental testing. Taken together, this has resulted in many in vitro skin culture models being developed [4–7]. Despite huge advancements in the field of skin tissue engineering over the last years, 3D in vitro skin models still show weaker barrier properties compared to human healthy skin [8] and do not contain skin appendages or many relevant immune cells and therefore do not mimic the complexity of the human skin. There is still an unmet need to develop skin models, which closely represent human skin physiology for hazard assessment and drug efficacy testing.

**Fig. 1:**
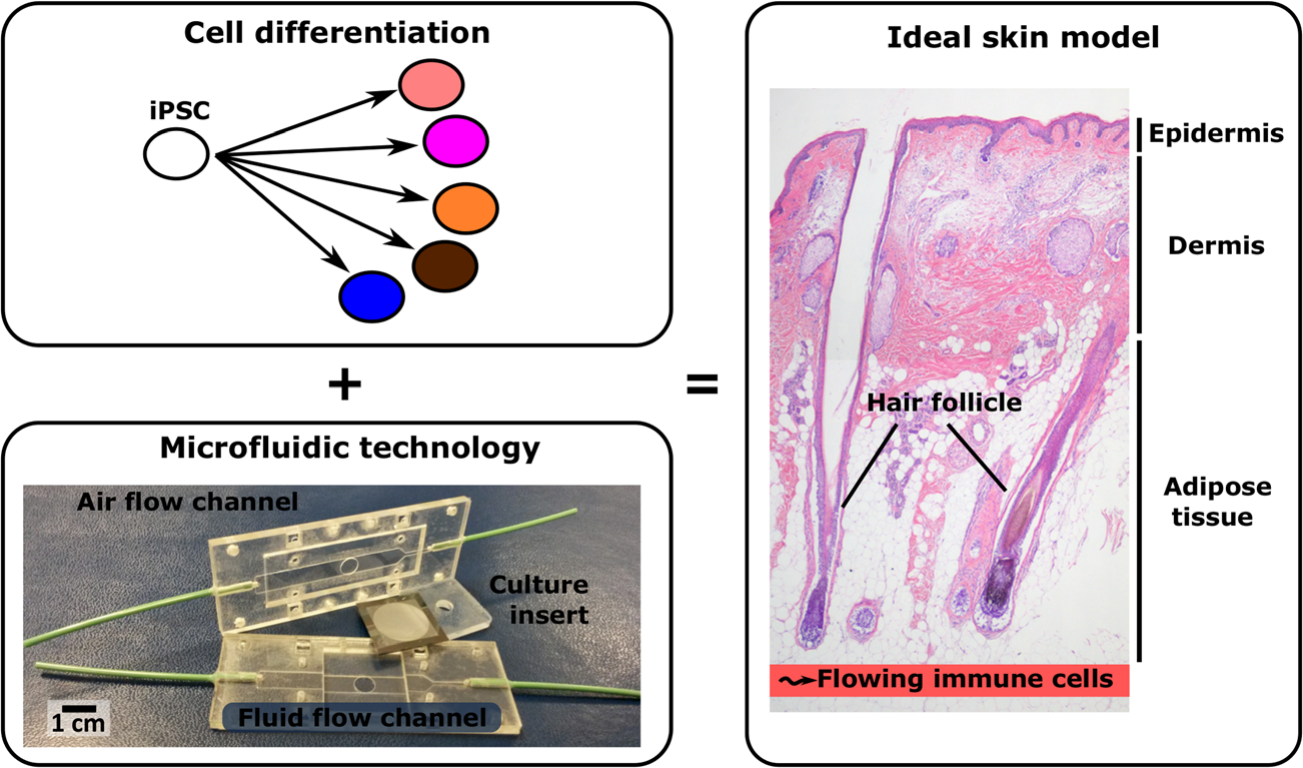
The combination of a microfluidic device (migration and immigration of immune cells and controlled environment) and iPSCs (all skin cells eg. fibroblasts, keratinocytes, melanocytes, dermal papillae cells, endothelial cells, adipocytes from same iPSC donor so no rejection) form the basis for the next generation skin models. Such an immunocompetent in vivo-like skin model containing three skin layers (epidermis, dermis and adipose tissue) and appendages would be an alternative to animal testing in toxicology assessment and drug testing

Our skin consists of three distinct compartments: epidermis, dermis and subcutaneous adipose (fat) tissue. Immune cells, e.g. Langerhans Cells, patrol the epidermis to initiate an immune response when the skin barrier is breached. Furthermore the skin is linked to the systemic circulation via micro-capillaries in the dermis and adipose tissue. The major appendage transversing all three compartments of the skin is the hair and therefore the hair shaft can be considered as a gatekeeper for the epidermis and a major route of penetration for anything coming into contact with the skin, as well as having a role in temperature regulation and personal appearance. Next to this, other skin appendages (e.g. sebaceous gland, sweat gland, arrector pili muscle) and nerves also have important roles in maintain skin integrity and homeostasis. The ideal in vitro 3D skin model would contain the complete package (Fig. 1).

Currently mainly primary cells and cell lines are used to construct skin models. Induced pluripotent stem cells (iPSC) may be an alternative source of cells used in skin tissue engineering, specifically for immune competence, appendages and inter-organ modeling (Fig. 1). Another significant advancement for 3D skin models may be achieved from the field of organ-on-chips, which enable more physiologically relevant conditions to be incorporated into skin models (Fig. 1). Organ-on-chip is a promising technology for drug development, substance testing and reduced animal testing [9, 10]. Organ-on-chip is a combination of microsystems technology and cell/tissue biology aimed to create more physiological relevant organotypic models [11, 12]. This is achieved by better mimicking the 3D microenvironment through microsystems engineering. The microsystems enable integration of channels and actuators, which provide control of medium flow, gradients of substances and mechanical loads. For skin-on-chip this could possibly lead to controlled 3D organization of skin layers and appendages, vascular microfluidic control of nutrients, waste products, immune active molecules and immune cells as well as the control of physical environmental factors (e.g. temperature, force, gas). The organ-on-chip technology may also allow integration of sensors for real-time readout of biomarkers. Additionally, in combination with iPSCs it is envisioned that different organ-models can be created and combined to create a patient specific multi-organ-on-chip model as a highly advanced tool for drug development [4–6]. Here we discuss the state of the art with regards to skin-on-chip models and cell sources (primary, cell line, induced pluripotent stem cells) that may enable the next step to be taken in skin disease modeling, substance testing, and ultimately personalized medicine.

### State of the art Primary Cell Models

Already for a long time, primary cells have been used to construct healthy and disease skin models. One of the main problems with using primary skin cells is that they have a limited number of population doublings and undergo senescence. Below we will focus on the state of the art of human healthy tissue engineered 3D in vitro skin models.

Besides the numerous in house developed skin models*,* nowadays skin models are also commercially available. Therefore advanced knowledge of cell culture techniques is no longer required and researchers and industry can make use of standardized skin models (Table 1). To date, the simplest available 3D skin model is an in vitro (pigmented) reconstructed epidermis from keratinocytes. This model represents the barrier function of the stratum corneum and is used for risk assessment. A slightly more complex in vitro skin model consists of a reconstructed epidermis on a fibroblast-populated dermis. Next to the barrier function of the stratum corneum there is to a certain extent cross talk between keratinocytes and fibroblasts. However, to date, these commercially available skin models do not contain endothelial cells, immune cells, adipose tissue or skin appendages and the barrier properties are reduced compared to in vivo human skin [13]. Therefore they are of limited physiological relevance for risk assessment and testing mode of action of novel actives.

**Table 1:** An overview of tissue-engineered 3D skin models from human primary cells and their limitations

Model	Commercial available	Advantages/disadvantages	Ref.
Reconstructed epidermis	Yes: EpiDerm™, EpiSkin™, SkinEthic™, epiCS® No: in house models	+: differentiated epidermis from keratinocytes -: only keratinocytes, no dermal compartment present or immune cells	[80, 81]
Pigmented Reconstructed epidermis	Yes: MelanoDerm No: in house models	+: pigmented differentiated epidermis from keratinocytes and melanocytes -: no living dermal compartment, immune cells, adipose tissue, appendages or blood vessels present	[27, 82]
Full-thickness skin models	Yes: EpiDerm-FT, Phenion-FT, LabSkin No: in house models	+: differentiated epidermis on fibroblast-populated dermis -: no immune cells, adipose tissue, appendages or blood vessels	[83–86]
Three layered skin model	No: in house models	+: differentiated epidermis on fibroblast-populated dermis on a adipocyte /ASC populated hypodermis -: no immune cells or appendages	[16–18]
Full-thickness skin model containing EC	No: in house models	+: differentiated epidermis on fibroblast and endothelial cell (show vessel like structures) populated dermis -: no immune cells, adipose tissue, appendages or perfused blood vessels	[14, 15]
Skin equivalent with integrated Langerhans Cells	No: in house model	+: pigmented skin model containing functional MUTZ-3 derived Langerhans -: no adipose tissue, appendages or blood vessels	[19, 20]

*ASC* adipose tissue-derived mesenchymal stromal cells

On the other hand several in house models are described in which endothelial cells, adipose tissue layer and immune cells have been added to full-thickness skin models (Table 1). Next to fibroblasts, endothelial cells have been added to the dermal compartment of a skin model where they form vessel-like structures [14, 15]. However, these skin models lack a functional perfused vasculature, limiting clinical and research applications. A third skin layer containing adipocytes (adipose tissue) has been added to the full-thickness skin models [16–18]. These skin models with a hypodermis containing adipocytes showed better epidermal differentiation and basal membrane protein expression than two layered skin models [18]. This model may be useful for introducing hair follicles, which lie partly in the adipose tissue. Until now only one reproducible skin equivalent with functional integrated immune cells (Langerhans Cells) has been described [19, 20]. The Langerhans Cells (MUTZ-3 derived) are able to initiate an innate immune response upon topical allergen or irritant exposure in a similar manner to native skin. The next stage in development of this model would be to introduce T-cells in order to investigate adaptive immune responses, like T-cell priming and sensitization.

An improvement of the current in vitro primary skin models may be achieved in the future by using microfluidic culture devices which may enable more physiologically relevant exchange of immune cells, a controlled environment and an increased barrier function. However until now only a few in vitro tissue-engineered 3D skin model using primary cells in a microfluidic device have been described (Table 4). For example Groeber and colleagues recently described the first in vitro full-thickness skin model with a perfused vascular network [21]. In vitro tissue-engineered 3D skin models using primary cells in a microfluidic device are extensively discussed below in the section: state of the art skin-on-chip models.

Another emerging field which may improve the complexity of skin models is 3D bioprinting [22–24]. 3D bioprinting provides a fully automated and advanced platform that facilitates the deposition of multiple types of skin cells and biomaterials in similar way to native human skin. Nowadays, most printed skin models consist of a epidermal layer on a fibroblast-populated dermis [25]. However more complexity may be added by printing vascular structure or a controlled environment for niche cells (eg. hair follicle, sweat glands) which can direct cell and tissue level functions [24]. For example Lui et al. showed a 3D printed matrix as the restrictive niche for direct sweat gland differentiation of epidermal progenitors into glandular morphogenesis in vitro [26]. In the future despite challenges of fabricating completely functional skin constructs, 3D bioprinting may facilitate comprising additional cell types and biomaterials to enhance the similarity to native human skin.

### State of the art Cell Line Models

In order to overcome the limitations of using primary cells, immortalized skin cell lines can be used. There are different ways to overcome cell senescence leading to an immortalized cell line e.g. spontaneous immortalization, overexpression of telomerase and/or telomerase reverse transcriptase (TERT), inactivation of p16^INK4A^ and p14^ARF^, retroviral transduction of HPV E6 and E7 (leads to inactivation of p53 and pRb) or simian virus 40 (SV40), and treatment with Rho kinase inhibitor (Y-27632) [27, 28]. The advantages of cell lines are the continuous availability, long-life span and reproducibility.

Several organotypic skin models, e.g. skin equivalents and skin-on -chips, with cell lines have been developed and are summarized in Tables 2 and 4 respectively. In short, for the epidermal component of a skin equivalent different keratinocyte cells have been used e.g. HPV-16 and HPV-18 immortalized keratinocytes [29, 30], HaCaT [31–34], near-diploid spontaneous immortalized human keratinocytes (NIKS) [35–37], hTERT immortalized keratinocytes [38, 39], cdk4 overexpression [40, 41], squamous cell carcinoma (SCC) [42, 43], Y-27632 immortalized keratinocytes [44, 45] and H9 hES differentiated cells [46]. The dermal component often consists of primary fibroblasts. The different immortalization methods and culture methods lead to a variation of the quality of the skin constructs regarding epithelial differentiation, stratification and barrier function. The HPV and SCC cell lines have (pre)malignant characteristics, whereas the NIKS and hTERT immortalized cell line constructs resembles the normal human skin [35, 38, 39]. Other cell line skin equivalents show deficiencies in differentiation and stratification (Table 2). In conclusion, the most optimal and reproducible cell line skin model resembling the healthy human skin which consists completely of cell lines and with extensive characterization (IHC, EM and secreting proteins) is the hTERT immortalized skin equivalent model in which both the keratinocytes as well as the fibroblasts are hTERT immortalized [39]**.**

**Table 2:** An overview of tissue-engineered 3D skin models from cell lines and their limitations

Epidermal component	Dermal component	Advantages/disadvantages	Ref.
Keratinocytes	Fibroblasts		
HPV-16 & HPV-18 immortalized human foreskin KCs	human foreskin dermal Fbs (primary)	+: bovine collagen matrix -: 1 component cell line -: no markers; disorganized and poorly differentiated (premalignant characteristics)	[29]
HPV (CIN 612-9E cells)	J2 3 T3 murine feeder cells (subclone)	+: L1 major capsid protein: SG & SC (IHC) +: rat tail collagen I matrix -: Fbs murine origin	[30]
HaCaT cells (spontaneous)	human dermal Fbs (primary)	+: K14, K1/10, INV, TGase, FIL, K2e & LOR (IHC) +: rat tail collagen I & DED matrix -: 1 component cell line -: prolonged culture time → epiboli-like configuration	[31]
HaCaT cells (spontaneous)	MRC-5 (human fetal lung Fbs) (spontaneous)	+: K1, INV, TGase1, K14 & LOR (IHC, WB and EM) +: collagen I matrix -: lung derived Fbs	[32]
NIKS (immortalized near-diploid human KC cell line; spontaneous)	human neonatal Fbs (primary)	+: morphology, K14 & FIL (IHC and EM) +: collagen I matrix -: 1 component cell line	[35–37]
hTERT-immortalized foreskin KCs	human foreskin Fbs (strain B256)	+: HE and K13 (IHC) +: collagen matrix -: 1 component cell line	[38]
hTERT-immortalized foreskin KCs	hTERT-immortalized foreskin Fbs (BJ-5ta)	+: morphology, K5, K10, INV, LOR, COLIV, LAM5, VIM & COLIII (IHC and EM) +: collagen/elastin matrix	[39]
Cdk4 overexpressing (and TERT immortalized) foreskin KCs	BJ normal human newborn foreskin Fbs (spontaneous)	+: K14, p63, INV, COLIV, LAM5 & VIM (IHC) compared with NS +: collagen matrix	[40, 41]
SCC-12B2; SCC-13 (spontaneous?)	normal human dermal Fbs (primary)	+: K10, K16, K17, Integrin β4, LAM332, INV, Axl & COLIV (IHC) (malignant characteristics) +: rat tail collagen matrix -: 1 component cell line	[42, 43]
Y-27632 (Rho kinase inhibitor) immortalized KCs	J2 3 T3 murine feeder cells (subclone)	+: rat tail collagen I matrix -: deficiencies in differentiation and stratification -: Fbs murine origin	[44]
Y-27632 (Rho kinase inhibitor) immortalized KCs	-	+: K14, K10, INV, TGase 1, LOR, FIL & LCE2 (IHC) +: DED matrix -: only epidermal component; no Fbs and matrix	[45]
H9 hES cells (differen-tiated to epithelial cells)	normal human Fbs (primary)	+: p63, K10, INV & FIL (IHC) compared with NS +: rat tail collagen matrix -: 1 component cell line	[46]

*Axl* transmembrane receptor tyrosine kinase, *COL* collagen, *DED* de-epidermized dermis, *EM* electron microscopy, *ES* embryonic stem cells, *Fb* fibroblast, *FIL* filaggrin, *HFKs* human foreskin keratinocyte, *HPV* Human papilloma viruses, *IHC* immunohistochemical staining, *INV* involucrin, *K* keratin, *KC* keratinocyte, *LAM* laminin, *LCE2* late cornified envelope 2, *LOR* loricrin, *NS* normal skin, *SC* stratum corneum, *SCC* squamous cell carcinoma, *SG* stratum granulosum, *TGase 1* transglutaminase 1, *VIM* vimentin, *WB* Western Blot

Functionality was tested for some organotypic skin models, ranging from skin inflammation and skin edema [33], skin barrier function [34], cytotoxicity testing, effect of ectopic gene expression [32, 36, 37, 40], wounding [39, 41], secretion of inflammatory mediators [33, 39] to an in vitro psoriasis model [45]. Hence, these organotypic skin models can be used for testing the effect of therapeutics and/or chemicals. However it should be noted that there is limited information available describing the integrity of the basement membrane and barrier function of these models (Table 2). Another limitation is the fact that until now most of the organotypic skin models only consist of keratinocytes in combination with fibroblasts (or keratinocytes only). Addition of other cell types, which are present in human skin, like endothelial cells, immune cells and hair follicles will improve the models in the future. One general limitation when using cell lines is that these models can not represent patient variation within a disease and therefore have limited use for personalized medicine approaches or for investigating variation within a population.

### State of the art Induced Pluripotent Stem Cells (iPSC) Models

IPSC might be the future of tissue-engineered skin models because iPSC potentially can be differentiated into unrestricted numbers of all cell types of the skin. These iPSC are derived from adult somatic cells via reprogramming with ectopic expression of reprogramming factors (e.g. the combination of Oct3/4, Sox2, c-Myc and Klf4) [47]. The expression of these reprogramming factors leads to the suppression of genes responsible for differentiation and to the expression of genes and epigenetic changes that sustain pluripotency, hereby reverting the cells to a pluripotent state. Initially this reprogramming was done by retrovirus-mediated transfection [48]. This and several other transfection methods have the risk of integrating the retrovirus into the genome. Therefore new delivery methods were developed using genome integrating-free approaches (e.g. protein, mRNA, episomal vector) [47]. These integrating-free methods can be used to obtain high quality iPSC, but are very inefficient compared to viral integrating methods. Also the choice of the somatic cell type influences reprogramming efficiency. IPSC can be generated from various tissues; skin, adipose tissue, cord blood, peripheral blood and urine [49]. From skin tissue, keratinocytes, melanocytes, fibroblasts and dermal papilla cells can be used to generate iPSC. Skin-derived fibroblasts are most often used due to their simple culture conditions and easy collectability.

IPSC can be differentiated in a wide variety of cells, including keratinocytes [50], fibroblasts [51], melanocytes [52], endothelial cells [53] and smooth muscle cells [54], leading to the potential use of iPSC-derived skin cells in skin tissue engineering for clinical applications and in vitro skin models mimicking healthy and diseased skin. The simplest available iPSC-derived healthy skin model is an in vitro reconstructed epidermis from iPSC-derived keratinocytes [55, 56]. This model showed similar differentiation, stratification and barrier-permeability compared to primary healthy epidermal model. Several full-thickness skin models are described containing iPSC-derived fibroblasts and/or keratinocytes (Table 3). Gledhill and colleagues further improved the complexity of this model by introducing iPSC-derived melanocytes into a reconstructed epidermis on a fibroblast-populated dermis [57]. This fully iPSC-derived 3D skin model showed similar morphology, differentiation and stratification compared to primary healthy skin model. The functional iPSC-derived melanocytes localized to the basal layer of the epidermis, extended dendrites into the suprabasal layers of the epidermis and produced melanin, which could be internalized by iPSC-derived keratinocytes [57]. Recently iPSC-derived endothelial cells were incorporated into a human primary skin model in a microfluidic device [58]. This perfused skin model showed a vascular network with an endothelial barrier function and normal skin barrier. The major drawback of the model is that the keratinocytes and fibroblast are not iPSC-derived, but it shows that increasing the complexity of skin models using iPSC is realizable.

**Table 3:** Overview of skin models from induced pluripotent stem cells

Source iPSC	Keratinocytes	Fibroblasts	Extra cell	Advantage/disadvantage	**Ref.**
Foreskin Fb; transfected with mRNA cocktail of c-MYC, SOX2, OCT4, KLF4, and LIN28	Similar p63, ITGB4, K14 protein expression as neonatal KC	None		+: compared to primary healthy epidermal model; Permeability: TEER; Morphology: HE; DEJ: LAM; SB/ suprabasal: K14; Suprabasal: K10, INV; SG: FIL, TCHH, LCE2B; SC/SG: LOR; Cell-cell junction: DSC1 -: no dermal compartment (transwell), Fbs, immune cells, EC or appendages	[55, 56]
Foreskin Fb; retroviral transduction of c-MYC, SOX2, OCT4, KLF4, (Nanog*)	None iPSC derived (foreskin) (SE) or not used (DE)	Similar CD10, CD13, CDz44, CD73, CD90, CD166, COLI, COLII, PDGFRb expression as foreskin Fb COLIII, FN and tenascin C are different expressed		+: compared to primary skin model; Morphology: HE; DEJ: COLIV +: collagen 1 matrix (SE)/ self-assembly approach (DE) -: KC not iPSC derived -: no immune cells, EC or appendages	[51, 87]
Foreskin Fb; retroviral transduction of reprogramming factors; SOX2, OCT3/4, KLF4	Similar K5, K8, K14, K15, K19, INV, FIL, p63, ITGB4, ITGA6 and E-Cad expression as foreskin KC	?		+: compared to primary skin model; Morphology: HE; SB/ suprabasal: cytokeratin, K14, K15 -: no clear methods construction skin model -: no immune cells, EC or appendages	[65]
Foreskin Fb; retroviral transduction of reprogramming factors; c-MYC, SOX2, OCT4, KLF4	Similar p63, DSG3, ITGB4, lam5, K14 K5 and COLVII protein expression as foreskin KC	Similar CD10, CD44, CD73, CD90, P4Hb, COLI, COLVII expression as foreskin Fb	MC: Similar SOX-10, MITF-M, gp-100 and melanin expression as foreskin MC	+: compared to primary healthy skin model Morphology: HE; DEJ: LAM5, COLVII; Suprabasal: K1; SC/SG: LOR +: functional iPSC-derived melanocytes +: type 1 collagen matrix -: no immune cells, EC or appendages	[50, 57, 88]
Foreskin Fb; episomal transfection with L-MYC, SOX2, OCT3/4, KLF4	None iPSC derived (foreskin)	None iPSC derived (foreskin)	EC: VE- cadherin and CD31 expression Formation of tube-like structures on matrigel	+: compared to skin model with HUVEC Morphology: HE; Permeability endothelial barrier function; SB/ suprabasal: K14; Suprabasal: K10; SC/SG: LOR; EC: CD31 +: functional iPSC-derived EC +: type 1 collagen matrix -: KC and Fb are not iPSC derived -: no immune cells or appendages	[58]

*Nanog in combination with other reprogramming factors is used in one of two generated iPSC lines; *COL* collagen, *DE* dermal equivalent, *DEJ* dermal epidermal junction, *DSC1* Desmocollin 1, *DSG3* Desmoglein-3, *EC* endothelial cell, *E-Cad* E-cadherin, *Fb* fibroblasts, *FIL* filaggrin, *FN* fibronectin, *gp-100* glycoprotein 100, *HE* Hematoxylin and eosin, *HUVEC* human umbilical vein endothelial cells, *INV* involucrin, *iPSC* induced pluripotent stem cell, *ITGB4* Intergrin beta 4, *ITGA6* Intergrin alpha 6, *KC* keratinocytes, *K* keratin, *KLF4* Kruppel-like factors 4, *LAM* laminin, *LCE2B* late cornified envelope 2B, *LOR* loricrin *MC* melanocytes, *MITF* Microphthalmia-associated transcription factor, *OCT4* Octamer binding transcription factor 4, *PDGFRb*, platelet-derived growth factor receptor-b, *P4Hb* Prolyl 4-Hydroxylase Subunit Beta, *SB* stratum basal, *SC* stratum corneum, *SE* skin equivalent, *SG* stratum granulosum, *SS* stratum spinosum, *SOX2* SRY (sex determining region Y)-box 2, *TCHH* Trichohyalin, *TEER* transepithelial electric resistance, *VE-cadherin* vascular endothelial cadherin

The next step would be to introduce iPSC-derived immune cells and appendages in the iPSC derived skin models. It has already been shown that iPSC can be differentiated into hematopoietic stem cells and their progeny. The generation of truly functional skin immune cells (e.g. Langerhans cells) is less clear. However during the last years it is shown that iPSC can be differentiated into several types of immune cells; T-lymphocytes, macrophages, granulocytes, erythrocytes and dendritic cells [59–62]. More research is needed to look into the differentiation of iPSC towards skin specific Langerhans cells. Hair follicle cells (dermal papilla and keratinocytes [63, 64]) are used to generate iPSC but the generation of hair follicle cells from iPSC is less described. To our knowledge no reports exist which describe the generation of dermal papilla cells from iPSC. Yang and colleagues show that human iPSC that were differentiated into epithelial stem cells expressing CD200 and ITGA6, are capable of generating all hair follicle lineages in mice including the hair shaft, and the inner and outer root sheaths keratinocytes in skin reconstitution assays in mice [65]. Combined with human dermal papilla cells, when dermal papilla cells can be generated from iPSC, these epithelial stem cells expressing CD200 and ITGA6 might possibly in the future be used to construct a fully human iPSC derived in vitro skin models containing ‘hair follicles’. Next to hair follicle cells, the human epithelial stem cells expressing CD200 and ITGA6 can be differentiated into sebocyte-like cells under sebocyte differentiation conditions [65]. Whether these sebocyte-like cells generate a sebaceous gland is not shown. However, generation of skin appendages in primary-based skin equivalents is also a research area in its infancy. Much is still to be learned to successfully culture appendages in vitro.

Next to generating healthy human skin models, iPSC derived from somatic cells containing a genetic mutation can be used to generate genetic diseased skin models. Already in 2011, Itoh and colleagues differentiated iPSC derived from patients with recessive dystrophic epidermolysis bullosa (RDEB) into keratinocytes [50]. The 3D skin equivalents constructed with the RDEB iPSC derived keratinocytes showed no expression of collagen VII similar to skin of patients with RDEB. Such skin disease models can be used as an in vitro model to study disease mechanism and/or to test novel (patient-specific) drugs. Furthermore, due to the possibility to isolate iPSC from blood or urine, unlimited genetic in vitro skin disease models could be made from patients from whom material-extraction is limited or harmful. Also for patients with limited healthy donor skin left (eg. large total body surface area burn wounds) iPSC from blood or urine give the possibility to make unlimited autologous skin grafts. Ultimately, for genetic diseases that lack adequate treatment, gene correction of the underlying genetic abnormality in patient specific diseased iPSC, e.g. through homologous recombination or Zinc Finger nuclease [66], can provide an unlimited source of immunologically matched cells to treat a patient clinically. However more research is needed to determine the stability, and safety of iPSC-derived therapies [67, 68].

A drawback of using iPSC is that the reprogramming and the subsequent verification of iPSC pluripotency are manually labor intensive, limiting the throughput time, scale and reproducibility. Recently though, the first high-throughput conversion of skin biopsies into iPSCs and differentiated cells with minimal manual intervention by a robotic platform has been described [69]. The authors demonstrate that automated selection can result in high-quality, more controlled and stable iPSCs. This robotic platform has the potential to increase the use of iPSCs.

Overall, iPSC-derived skin cells are an unlimited source of cells, which can be used to construct in vitro healthy and diseased skin models. Next to skin models, other organs can be generated from the same iPSC donor. In the future this may lead to the possibility to connect immunologically matched organs in vitro. For clinical applications iPSC might be promising, but more research is needed to produce high quality iPSC and their safety in vivo.

### State of the art Skin-on-Chip Models

Skin-on-chip is one of the various organ-on-chip models that is under development, pursuing the ambition to create more physiologically relevant exchange of immune cells, controlled environment and increased barrier function (Table 4). The Marx-group has developed a multi-organ-on-chip for skin, hair and other tissues using a multi-chamber microfluidics device with integrated pumping of sub-ml volumes. The device integrates tissue engineering and substance testing, enabling regular (immuno)histological analysis and supernatant extraction for metabolite analysis to be performed [70–73]. Despite demonstrating the potential of organ-on-chip for long term culturing and repeated dose testing of substances, there is room for improvement with regards to tissue complexity, differentiation, viability and barrier function before the model can be fully implemented to study skin disease, skin biology and personalized medicine. Wufuer and co-workers report a simplistic model for substance penetration in skin, by stacking a bi-layer of keratinocytes-fibroblasts and endothelial cells-fibroblasts between three microfluidic channels [33]. The immune competent keratinocyte-on-chip by Ramadan and Ting describes the interaction between HaCaT KC-cell line and U937 monocytic cell line in a bi-channel microfluidic device [34]. This is the only skin-on-chip model including an immune component. The authors demonstrate its potential for substance testing using LPS and UV stimulation. The effects are assessed by trans-epithelial electrical resistance (TEER) measurement and magnetic bead immune assay, both of which are integrated into the device. Abaci and co-workers demonstrated a simple full-thickness skin equivalent for percutaneous penetration into the medium [74]. The model included gravity driven microfluidic channels to collect penetrated substances in small volumes, enabling physiologically based pharmacokinetics modeling.

**Table 4:** Overview of organ-on-chip models of skin

Model (*source*)	Skin equivalent / Cell types	Application achievement	Advantages/disadvantages	Ref.
KC-on-chip *(primary)*	NH neonatal KC	High viability at near-confluency	+: microfluidic flow over cells potentially for high throughput screening -: monolayer culture	[89]
Immune competent KC-on-chip *(cell line)*	HaCaT KC CL U937 monocyte CL	Monocyte/KC interaction on chip under LPS or UV stimulation	+: on-chip TEER measurement for continuous tight junction +: on-chip magnetic bead immune-assay for il-6, il-1β +: 17d KC viability +: 100% cell line -: non-organotypic	[34]
Skin-on-chip *(cell line)*	HaCaT KC CL HS27 FB CL HUVEC	Multi monolayer skin inflammation /edema model	+: mimics KC-Fb interaction Fb-EC interaction +: simple model for paracrine signaling -: non-organotypic	[33]
Skin-on-chip *(primary)*	EpidermFT™ (FT,NH KCs & Fbs), with/ without ex vivo *s*ubcutaneous tissue	7 day Tissue maintenance through dynamic perfusion	+: use of biopsies and SE +: use of adipose tissue -: no endothelial barrier to flow -: no mechanical effects of flow -: high frequency medium change	[70]
Skin-on-chip *(primary)*	Biopsies of FT SE, of human foreskin KCs & Fbs, COL1 based	3 week PBPK/PD testing of skin equivalent	+: simple pumpless microfluidics +: transdermal transport model -: markers (Ki67, K1, K14, Loricrin) -: only KC and Fbs	[74]
Vascularized skin-on-chip *(primary)*	NH dermal Fbs, NH KCs, HUVECs, COL1 based	10 day perfusion of vascularized FT skin equivalent	+: percutaneous absorption of substances into vasculature +: non-micro culture conditions +: direct EC-ECM interaction -: limited characterization of epidermal markers (K10, K15)	[75]
Vascularized skin-on-chip *(primary/iPSC)*	NH dermal Fbs, NH KCs, HUVEC and iPS based ECs, COL1 based	In vivo Neovasculari-zation of vascularized FT skin equivalent	+: iPSC based endothelial cells +: non-micro culture conditions +: direct EC-ECM interaction +: vascularization improves basal layer -: long culture period (21d) before application of flow	[58]
Skin/hair-follicle-on-chip *(primary)*	Biopsy from ex vivo prepuce Skin hair follicular unit extracts	14 day ex vivo tissue maintenance through dynamic perfusion	+: co-culture of ex vivo skin and hair follicle in separate wells +: perfusion reduces tissue degradation -: no endothelial barrier to flow -: no mechanical effects of flow -: high frequency medium change	[70]
Skin/liver-on-chip *(primary)*	HepaRG hepatic CL, HHSCs, HDMEC Juvenile prepuce skin biopsies	28 day cultivation and 14 day repeated dose substance testing	+: liver-skin cross talk demonstrated +: 14 day repeated dose testing +: endothelialized microfluidics -: usage of primary skin biopsies -: high frequency medium change	[71, 72]
Skin/liver/ kidney/gut-on-chip *(primary)*	Juvenile prepuce skin biopsies, EpiIntestinel™, HepaRG hepatic CL, HHSC, NPTCL RPTEC	28 day 4-organ co-culture, separate microfluidics for surrogate blood and excretory flow	+: 4-organ co-culture for ADME +: two fluidic circuits resembling blood flow and kidney-excretion -: usage of primary skin biopsies -: lack of model blood-skin barrier -: high frequency medium change	[73]

*ADME* absorption, distribution, metabolism and excretio, *CL* cell line, *COL* collagen, *EC* endothelial cell, *ECM* extra cellular matrix, *Fb* Fibroblast, *FT* full-thickness, *HDMEC* human dermal microvascular endothelial cell, *HHSC* human hepatic stellar cell, *HPTCL* human proximal tubule cell line, *HUVEC* human umbilical vein endothelial cell, *KC* Keratinocyte, *LPS* lipopolysaccharide, *NH* normal human, *RPTEC* human proximal tubule cell line RPTEC/TERT-1, *SE* skin equivalent, *TEER* trans epithelial electrical resistance

Further improvement of physiological relevance is being achieved through perfusable vascularized full-thickness models. Abaci and co-workers realized a perfusable vascularized full-thickness skin equivalent with HUVEC and notably iPSC derived endothelial cells [58]. Micro-structured molding of dissolvable alignate-gel was used to create the vasculature in a contracted collagen-based dermis. This model demonstrated improved neovascularization in a murine graft-model. Finally, Mori and co-workers developed a perfusable full-thickness skin equivalent using HUVEC lined nylon wires within the dermal compartment which also prevented extracellular matrix (ECM) contraction [75]. Although only a limited assessment of epidermal and basement membrane biomarkers was performed, the authors did demonstrate percutaneous penetration into the endothelialized tubes, a promising result for drug testing. Equally promising for skin disease modeling is the direct endothelial cell-matrix interaction in this model since it mimics the blood vessel-tissue barrier more closely. Another particularly interesting model is that described by Groeber and colleagues. They developed the first in vitro full-thickness skin model with a perfused vascular network, which was based on a decellularized segment of porcine jejunum (containing a conserved vascular structure), primary endothelial cells, fibroblasts and keratinocytes and a tailored bioreactor system [21]. Although these reported vascularized models lack cellular components such as pericytes and immune cells, they have the potential to study the interaction of peripheral blood derived immune cells with different layers of the skin.

Taken together, these reports demonstrate the potential of skin-on-chip models mainly for substance testing. Future improvements can be expected to obtain healthy and diseased skin models for disease progression/remission modelling, and repeated dose toxicity testing. Technological improvements that would greatly facilitate disease modeling and personalized medicine turn-around time are integration of sensors, optimized designs for mass-fabrication, user-friendly handling and bubble-free flow control [9, 76–79]. The on-going interdisciplinary collaboration between microsystems and biomedical researchers, to optimize desired read-outs, tissue-engineering methods, physical and manufacturing possibilities will certainly yield solutions. Improvements to the microphysiological relevance of skin-on-chip models may be achieved with strategies that mainly entail the biological context (ECM, cell types), sustained cultures (culture period, culture medium changes), mechanical cues (stretch, ECM stiffness) and chemical cues (medium composition, air composition) [79]. Optimization of ECM composition and stiffness could improve contraction, which is usually unreported but may pose problems to maintaining a consistently leak-free fluid-tissue-air barrier in skin-on-chip. Airflow and gas composition control in the air-exposed compartment could be used to improve differentiation, stratification and homeostasis, mimicking the normal outer environment of skin. Furthermore, controlling the strain on the tissue would enable understanding the role of mechanics in wound healing. Additionally, iPSC would greatly improve physiological relevance by including more cell types, as discussed in section “state-of-art iPSC models”: iPSC provide a source of cells to create all relevant cell types from limited amount of patient material.

## Conclusion/Summary

Skin disease modeling, substance testing, and ultimately personalized medicine would be enabled by an ideal in vitro 3D skin model containing vasculature, immune cells and appendages. Until now full-thickness skin models based on primary cells are most common, even available commercially. Commercial models have limited physiological relevance for risk assessment and testing mode of action of novel actives. Different in house skin models are improving relevance by incorporating endothelial, immune cells, adipose tissue and microfluidics technology. The drawbacks of using primary cells in full-thickness models are senescence, limited population doubling and reproducibility. Cell lines are advantageous in these respects, although these have only been demonstrated in organotypic skin models with keratinocytes and fibroblasts. Other skin cell types, will improve such models in the future, but these cell lines would not represent patient variation within a disease, limiting personalized medicine approaches. IPSC on the other hand could potentially be differentiated into unlimited amount of all skin cell types with healthy and diseased characteristics. Currently iPSC-based full-thickness models including keratinocytes, fibroblasts, melanocytes and endothelial cells, have been described. Improvements in iPSC based models involve enhancements to barrier and dermal properties and integration of iPSC derived immune cells and appendages. Drawbacks of iPSC are mainly in the logistics resulting low yield and reproducibility, high costs and clinical application, although lab-automation is being developed to improve these aspects. Skin-on-chip would increase physiologically relevance through exchange of immune cells, controlled environment and increased barrier function. Most reported skin-on-chip models demonstrate the potential of skin-on-chip models mainly for substance testing, some being perfusable vascularized full-thickness models. Improvements to skin-on-chip technology, i.e. microfluidics, availability, read-outs, and biology (i.e. cell types and matrices) are necessary for repeated dose toxicity testing or disease progression/remission modelling. Nonetheless, skin-on-chip and iPSC are advancing and their combination will lead to better healthy and diseased skin models and ultimately personalized medicine. However organotypic models based on primary cells and cell lines have their merits depending on their application. They will therefore likely maintain a relevant role, besides iPSC, in skin research.
